# Supplementary material for: Multi-class classification algorithms for the diagnosis of anemia in an outpatient clinical setting
Source: PLoS One. 2022 Jul 6;17(7):e0269685. doi: 10.1371/journal.pone.0269685 (PMC9258850; doi:10.1371/journal.pone.0269685)
Supplement: S1 Appendix — (DOCX) [file pone.0269685.s001.docx]

**Appendix 1**

Medical terminology**-**In this section we define the medical terms used in this paper.

**Anemia**

This is a medical health condition which reduces the haemoglobin concentration in the blood thus adversely affecting its oxygen carrying capacity. This disease can cause many complications such as reduced work capacity, reduced ability in carrying out daily activities, reduced cognitive ability and fatigue. A number of factors can cause anemia such as iron deficiency which is studied in this paper.

Complete Blood Count (CBC) test

This is the most widely used blood test to measure the overall health of a person and to detect a number of diseases that includes anemia.

Microcytic, Normocytic and Macrocytic

In the defined data set of CBC test parameters, we can classify anemia as microcytic, normocytic or macrocytic based on the value of the attribute MCV (Mean cell volume) as follows:

Microcytic: MCV < 80 fl.

Normocytic: MCV: 80 – 100 fl.

Macrocytic: MCV > 100 fl.

This helps in the further diagnosis of the patient.

Haemoglobin

Anemia is diagnosed on the basis of the value of the attribute Haemoglobin (HGB), which is a key attribute in the CBC patient data set. It is defined separately for men and women for the purpose of classifying anemia as mild, moderate or severe. This tabulation is shown as follows:

Men Women

Condition for Anemia: Condition for Anemia:

HGB < 13.0 g/dl. HGB < 12.0 g/dl.

Mild Anemia: Mild Anemia:

HGB: 11.0-12.0 g/dl. HGB: 11.0 – 12.0 g/dl.

Moderate Anemia: Moderate Anemia:

HGB: 8.0 – 11.0 g/dl. HGB: 8.0 -11.0 g/dl.

Severe Anemia: Severe Anemia:

HGB < 8.0 g/dl. HGB < 8.0 g/dl.

We see that the degree of anemia prevalence is defined on the basis of the haemoglobin concentration.

CBC TEST PARAMETERS

We have defined 11 attributes in the CBC test data set. These are now briefly defined.

Age: Age of the patient.

Gender: Gender of the patient.

MCV: Mean cell volume. This measures the average size of the red blood cells. Normal values are 80-100 fl.

MCH: Mean cell haemoglobin. This defines the average weight of haemoglobin per red cell. Normal levels are 28 – 33 pg(picograms).

MCHC: Mean cell haemoglobin concentration. This defines the average concentration of haemoglobin per erythrocyte – normal levels are 32 % - 36%.

RDW: Red cell distribution width. This is a quantitative measure of the uniformity of individual cell size – normal levels are 11% - 14%.

Platelet count: These are small elements formed in the red bone marrow that help to control bleeding. The number of platelets is the platelet count and iron deficiency anemia can elevate the platelet count. Normal levels are 150,000 – 350,000 / cubic mm.

WBC: White blood cells help in response to injuries and WBC measures the number of white blood cells in the body. Normal levels are 5-10 k/ul.

RBC: RBC counts how many red blood cells are present in the body. They contain haemoglobin that carries oxygen to the body’s tissues which need oxygen to function. Normal levels are 4.7-6.1 million RBC/microlitre for men and 4.7-5.4 million RBC/microlitre for women.

PCV: Packed cell volume is a measure of the fraction of blood that is made up of cells and is written as a % that represent the fraction of cells in blood.
