# Supplementary material for: Multi-class classification algorithms for the diagnosis of anemia in an outpatient clinical setting
Source: PLoS One. 2022 Jul 6;17(7):e0269685. doi: 10.1371/journal.pone.0269685 (PMC9258850; doi:10.1371/journal.pone.0269685)
Supplement: Attachment — Submitted filename: rebuttalletter.docx [file pone.0269685.s002.docx]

**Dr Rajan Vohra**

**Department of Computer Science**

**Liverpool John Moore’s university.**

Byom Street.

Liverpool.

L3 3AF.UK.

TO

**The Editor in Chief**

**PLOSONE**

1265 Battery Street, Suite 200
San Francisco, CA 94111
United States.

Dated 15^th^ December 2021.

Dear Sir or Madam

**Subject: Rebuttal letter accompanying revised manuscript.**

The authors would like to first and foremost thank the Editor, PLOS ONE and the reviewer, for taking out time and putting efforts in finding the shortcomings of the article and giving their supportive comments in making the article strong.

The comments/suggestions given by the reviewer are addressed one by one in the following section to the full of our knowledge and belief.

The following are the comments/suggestions given by the reviewer #1.

Major Comments:

1. On page 4, in line 2 of the 1th paragraph Data Analysis, More explanations are needed for feature selection techniques.

Resolution: The authors have provided detailed information about feature selection techniques and their techniques used for this research works.

2. On page 10, in line3-6 of the 2th paragraph Results and Discussion, LR is correct to achieve the highest accuracy and kappa value in table 5, but DT and MLP are the highest values in table 6 respectively. The sentence should be modified to fit the table.

Resolution: The author would like to thank reviewer for pointing out the mistake. It has been corrected.

3. On page 10, in Results and Discussion, the contents of the 4^th^ paragraph do not match Table 6.

Resolution: The contents of the 4^th^ paragraph has been updated according to the Table 6 values.

4. On page 10, in the Results and Discussion, there seems to be no clinical meaning in the line11-13 of the 5^th^ paragraph. Since the severe class is relatively small percentage of the dataset, when a SMOTE is used, the overfitting result is natural. In addition to MLP, other algorithms also achieved 100%, so it does not seem to be an ideal number.

Resolution: The authors agree with the reviewers comments. The reason behind getting this type of overfitting results is justified in the subsequent lines 13-15 of the said paragraph.

5. On page 10, in Results and Discussion, the contents of the 6^th^ paragraph do not match Table 8.

Resolution: The contents of the 6^th^ paragraph of the Results and Discussion has been updated according to Table 8.

6. On page 11, in Results and Discussion, lines 1 and 2 of the 9^th^ paragraph do not match Table 6.

Resolution: Relevant correction has been done as per the Table 6 to rectify the mistake made when writing the 9^th^ paragraph of Results and Discussion.

7. The criteria for classifying mild, moderate, and severe are not mentioned in the manuscript.

Resolution:The criteria for classifying an anemia into mild, moderate and severe is given at 1^st^ paragraph On page 4, of the Data Analysis.

Minor Comments:

1. It would be better if the designation of anemia and anaemia were unifield in the manuscript

Resolution: keyword anaemia is replaced with anemia in the manuscript.

2. On the page 1, in line 5 of paragraph abstract, rather than a simple description of dividing in to three classes, it seems necessary to explain more about the reason for dividing.

Resolution:The criteria for classifying an anemia into mild, moderate and severe is given at 1^st^ paragraph of the Data Analysis.

3. On page 1,In line 3 of paragraph Introduction,in front of ‘Anaemiaprevalence’, ‘,’ isrequired and space should be written.

Resolution: Done

4. On the page 1,In line 4 of paragraph Introduction,A space is needed in ‘whileanaemia’.

Resolution: Done

5. On the page 1,In line 10 of paragraph Introduction,Aspace is needed in ‘Anaemiasis’.

Resolution: Done

6. On the page 2,In line 1 of the 7^th^ paragraph Introduction,A space is needed in ‘analysiswhile’.

Resolution: Done

7. On the page 5, In line 9from the bottom of the paragraph Data Analysis, A space is needed in ‘Table 3shows’.

Resolution: Done

8. On page 8, It seems that Precision (Moderate) and Precision (severe) should be changed to recall (moderate).recall (severe).

Resolution: corrected

9. On page 8,It seems that true negative should be defined as true positive in precision formula

Resolution: corrected

10. On page 9, the exact definition or explanation of total accuracy and random accuracy in the formula for kappa should be included in the text.

Resolution: Done

11. On pages 12 to 14,In Table 5, Table 6, Table 7, and Table 8, it seems to indicate(%) in Accuracy.

Resolution: Done

12. On page 13, in the title of Table 7, it seems that Smote should be capitalized as SMOTE.

Resolution: Done

13. On page 14, in the title of table 8, ‘Smote fs’ seems to be written as SMOTE and feature selected’. If it is written as an abbreviation such ‘fs’, it may not be understood by readers.

Resolution: Done

14. On page 5, since the contents of fig 2 and Table 2 overlap, it would be better to use only one of them.

Resolution: Done

Yours Sincerely

**RAJAN VOHRA**
