# Supplementary material for: Multi-class classification algorithms for the diagnosis of anemia in an outpatient clinical setting
Source: PLoS One. 2022 Jul 6;17(7):e0269685. doi: 10.1371/journal.pone.0269685 (PMC9258850; doi:10.1371/journal.pone.0269685)
Supplement: Attachment — Submitted filename: rebuttalletter (2).docx [file pone.0269685.s003.docx]

**Dr Rajan Vohra**

**Department of Computer Science**

**Bolton University.**

A 676 Deane Road

Bolton.

BL3 5AB.UK.

TO

**The Editor in Chief**

**PLOSONE**

1265 Battery Street, Suite 200
San Francisco, CA 94111
United States.

Dated 1^ST^ April 2022.

Dear Sir or Madam

**Subject: Rebuttal letter accompanying revised manuscript.**

The authors would like to first and foremost thank the Editor, PLOS ONE and the reviewer, for taking out time and putting efforts in finding the shortcomings of the article and giving their supportive comments in making the article strong.

The comments/suggestions given by the reviewer are addressed one by one in the following section to the full of our knowledge and belief.

The following are the issues given by the reviewer.

Issues:

1. Please amend the manuscript submission data (via edit submission ) to include author Wasiq Khan and Celestine Iwendi

Resolution: done

2. Please include a separate legend for each figure in your manuscript.

Resolution: done

Yours Sincerely

**RAJAN VOHRA**
